# Interleukin-10 Genotype Correlated to Deficiency Syndrome in Hepatitis B Cirrhosis

**DOI:** 10.1155/2012/298925

**Published:** 2012-05-28

**Authors:** Qing-Ya Li, Zhi-Zhong Guo, Jian Liang, Wei Zhang, Lie-Ming Xu, Yue-Qiu Gao, Xiao-Su Wang, Dong-Ying Xue, Shi-Bing Su

**Affiliations:** ^1^Research Center for TCM Complexity System, Shanghai University of TCM, Shanghai 201203, China; ^2^Henan University of TCM, Zhengzhou, Henan 450008, China; ^3^Ruikang Hospital of Guangxi University of TCM, Nanning, Guangxi 530011, China; ^4^Longhua Hospital, Shanghai 200126, China; ^5^Shuguang Hospital, Shanghai 200021, China; ^6^Yueyang Hospital, Shanghai 200437, China; ^7^Putuo Hospital, Shanghai 200060, China

## Abstract

Traditional Chinese medicine (TCM) syndrome is an important basis for TCM diagnosis and treatment. As Child-Pugh classification as well as compensation and decompensation phase in liver cirrhosis, it is also an underlying clinical classification. In this paper, we investigated the correlation between single nucleotide polymorphisms (SNPs) of Interleukin-10 (IL-10) and TCM syndromes in patients with hepatitis B cirrhosis (HBC). Samples were obtained from 343 HBC patients in China. Three SNPs of IL-10 (−592A/C, −819C/T, and −1082A/G) were detected with polymerase chain-reaction-ligase detection reaction (PCR-LDR). The result showed the SNP-819C/T was significantly correlated with Deficiency syndrome (*P* = 0.031), but none of the 3 loci showed correlation either with Child-Pugh classification and phase in HBC patients. The logistic regression analysis showed that the Excess syndrome was associated with dizzy and spider nevus, and the Deficiency syndrome was associated with dry eyes, aversion to cold, IL-10-819C/T loci, and IL-10-1082A/G loci. The odds ratio (OR) value at IL-10-819C/T was 4.022. The research results suggested that IL-10-819C/T locus (TC plus CC genotype) is probably a risk factor in the occurrence of Deficiency syndrome in HBC patients.

## 1. Introduction

Hepatitis B virus (HBV) infection is a major health problem in China. It is one of the major causes of virus-related liver diseases such as liver cirrhosis (LC) and hepatocellular carcinoma (HCC) [1, 2]. There are 350 million HBV-infected people around the world, about 15–25% of who have the risk of dying of the HBV-caused LC and HCC [3]. During the course of HBV infection, liver is gradually damaged by this hepatotropic DNA virus, presenting a wide variety of clinical manifestations ranging from an asymptomatic carrier state to chronic hepatitis B (CHB), and to HBC, even to HCC [2, 4]. Five-year survival rate of patients with severe CHB and HBC is about 50% [5–7]. Whether chronic HBV infection would be developed to CHB or even LC, the process is affected by many factors such as hereditary susceptibility of patients.

DNA sequence and its variations are reflecting the human evolutionary process. SNP is a variation occurring when a single nucleotide in the genome (or other shared sequence) differs between members of a biological species or paired chromosomes in an individual [8]. It can help us to understand the occurrence and development of human diseases, and the response to drug therapy. Currently, it is known that at least 93% of human genes could present SNPs [9], and the SNPs have become the research focus of genetic susceptibility, and in the process of infectious diseases [10]. The previous studies have shown the correlation between the hereditary susceptibility of gene and liver disease, such as SNPs of aldehyde dehydrogenase 2 (ALDH2) and HCC [11], interleukin-2 (IL-2), IFN-*γ*, IL-10 and the infection of HBV, HCV, or HBV/HCV coinfection [12]. However, the studies of the correlation between genetic susceptibility and HBC are quite few.

The classification of disease is an important basis for the diagnosis of disease. In the LC, the phenotypes have been used to the classification, such as Child-Pugh classification, compensation and decompensation phase, hepatocellular function, and traditional Chinese medicine (TCM) syndromes. The TCM syndrome, also called ZHENG or TCM pattern, is the basic unit and the key concept in TCM theory. All diagnostic and therapeutic methods in TCM are based on the differentiation of ZHENG [13]. ZHENG is an abstraction of TCM theory, which can be seen as a simply assemblage of disease symptoms.

In this study, we investigated the correlation between the IL-10 gene SNPs and phenotypes, which include TCM syndromes, Child-Pugh classification, and compensation or decompensation phase in HBC patients.

## 2. Materials and Methods

### 2.1. Patients and Samples

In this study, 343 patients were recruited from Longhua Hospital, Shuguang Hospital, Yueyang Hospital and Putuo Hospital in Shanghai, the First Affiliated Hospital of Henan University of TCM and Ruikang Hospital in Guangxi in China. The patients were selected based on their age, gender, disease classification and area distribution (Table 1). All patients were Chinese yellow race. Blood samples were obtained from all subjects with informed consent and approved by ethic committee in respective hospitals, based on the Declaration of Helsinki. Each subject donated 3 mL peripheral blood samples, which were collected and stored at −80°C before DNA extraction.

### 2.2. Child-Pugh, Phase, and TCM Syndrome

The patients were divided into class A, class B, and class C according to Child-Pugh-Turcotte (CPT) score, and the CPT score was calculated by rating the following five parameters including serum levels of bilirubin and albumin, prothrombin time, ascites, and encephalopathy [14, 15]. According to clinical symptoms of patients and development of the disease, liver cirrhosis was divided into compensation phase and decompensation phase.

The clinical information of HBC patients such as symptoms and signs was collected from the above 6 hospitals, and then TCM syndromes were classified into Excess, Deficiency, and Deficiency-Excess syndromes by 3 TCM senior physicians according to the define of diagnosis, and TCM syndrome differentiation of liver cirrhosis [16]. The Excess syndrome was including Liver-qi stagnation syndrome, Damp-heat syndrome, and Blood stasis syndrome. The Deficiency-Excess syndrome was Damp abundance and spleen asthenia syndrome. The Deficiency syndromes were including liver-kidney yin deficiency syndrome and Yang deficiency of spleen and kidney syndrome.

### 2.3. DNA Extraction

Blood samples of all the patients were collected in K_2_EDTA tubes. Genomic DNA was selected from 1 mL peripheral blood of each sample, using the TIANamp Blood DNA Kit (Tiangen Biotech, Beijing, China). Subsequently, the DNA was stored at −80°C for following genotype analysis.

### 2.4. SNP Genotyping

Firstly, the genomic DNA extracted from clinical samples was subjected to a multiplex PCR (Invitrogen, Carlsbad, CA). Briefly, 1 *μ*L of genomic DNA was added to a 15 uL final volume containing 0.25 *μ*L per primer (10 *μ*mol/L) (prime sequence in Table 2), 0.2 *μ*L Taq enzyme, 1.5 *μ*L 10 × PCR buffer, 0.3 *μ*L dNTPs (2.5 mmol/L), 1.5 uL Mgcl2 and 10 uL deionized water. The reaction mixture was followed by a denaturation step at 94°C for 2 min and 35 cycles of amplification (94°C for 20 s, 60°C for 20 s, and 72°C for 40 s) with a final extension step at 72°C for 3 min through ABI 9600 (Applied Biosystems).

Secondly, LDR assays were carried out using conditions similar to those described elsewhere with slight modifications [17]. Briefly, the final volume containing 2 uL PCR products, 1 uL 10 × Taq DNA ligase buffer, 0.125 uL Taq DNA ligase (40 U/uL, New England Biolabs, Beverly, MA) and 0.01 *μ*L per probe (10 bp) and 6.845 *μ*L deionized water. LDR probes were designed by the Generay Biotechnology Company (probes sequence in Table 3). LDR mixtures were thermally cycled for 20 cycles of 30 s at 94°C and 3 min at 64°C through ABI 9600 (Applied Biosystems).

Lastly, the mixture of 1 *μ*L LDR product and 2 *μ*L loading Dye was followed by a denaturation step at 95°C for 3 min and was immediately put into ice-water. Then, the products were detected by ABI 3730XL DNA sequencer (Applied Biosystems).

Additionally, about 5% of the samples were randomly selected and retested by direct DNA sequencing in Shanghai National Biochip Research Center Laboratory, and the results were concordant with PCR-LDR.

### 2.5. Statistical Analysis

The data determined by the frequency of genotype obeyed the Hardy-Weinberg equilibrium (HWE) between the observed and expected genotype values. The correlation between genotypes and phenotypes was compared by the *X* ^2^ test. *P* < 0.05 was considered statistically significant in all tests. A binary logistic regression analysis was used for the evaluation of the independent effect of IL-10 SNPs on the TCM symptoms of HBC. Odds ratio (OR) and 95% confidence interval (CI) were rated.

## 3. Results

### 3.1. Characteristics of the Study Population

The frequencies of 3 SNPs loci of IL-10 were assessed in 343 HBC patients in China. The Hardy-Weinberg equilibrium (HWE) test showed that the distribution of these tested genotypes was not significantly different from the expected distribution (*P* > 0.05) (Table 1). The ages of patients ranged from 18 to 65 years old (mean ± SD, 49.57 ± 10.02). There were no significant differences of age and sex in gene polymorphisms in research object (*P* > 0.05). Males were 242 (70.55%) and females were 101 (29.45%). In Child-Pugh classification, class A, class B, and class C were 240 (69.97%), 75 (21.87%), and 28 (8.16%), respectively. There were 203 (59.18%) in compensation phase and 140 (40.82%) in decompensation phase.

### 3.2. Correlation between IL-10 Genotypes and Child-Pugh Classification and Compensation or Decompensation Phase in HBC Patients

As shown in Table 4, there was no significant correlation between IL-10 genotypes and Child-Pugh classification in HBC patients. It showed that the *P* value was greater than 0.05 between IL-10 genotypes (−592A/C, −819C/T, and −1082A/G) and class A, class B and class C of Child-Pugh classification, respectively. Also, there was no significant correlation between IL-10 genotypes (−592A/C, −819C/T, and −1082A/G) and compensation or decompensation phase, respectively (*P* > 0.05).

### 3.3. Correlation between IL-10 Genotypes and TCM Syndromes in HBC Patients

The correlation was analyzed between IL-10 genotypes (−592A/C, −819C/T, and −1082A/G) and TCM syndromes in HBC patients. As shown in Table 5, TC plus CC genotype of IL-10-819C/T was significantly different with TT genotype (*P* = 0.031) between Deficiency syndrome and other TCM syndromes. However, there was no significant correlation between IL-10-592A/C and −1082A/G genotypes and TCM syndromes (*P* > 0.05). It indicated that the patients with TC plus CC genotype of IL-10-819C/T may be appearance of Deficiency syndrome.

### 3.4. Correlation between the TCM Syndromes and Clinical Data and IL-10 SNPs in HBC Patients

To further clarify the correlation between Excess syndrome or Deficiency syndrome and clinical data and IL-10 SNPs in HBC patients, the binary logistic regression analysis was carried out. The analytic parameters were including age, gender, IL-10 SNPs loci (−592A/C, −819C/T, and −1082A/G), clinical symptoms and signs (fatigue, poor appetite, abdominal distension, backache, limp aching knees, dry eyes, dizzy, pruritus, yellow urine, aversion to cold, loose stools, spider nevus, ascites) and hepatocellular function parameters (ALT, AST, bilirubin and albumin, prothrombin time). The results showed that the Excess syndrome was associated with dizzy and spider nevus (Table 6), and the Deficiency syndrome was associated with dry eyes, aversion to cold, IL-10-819C/T, and −1082A/G loci (Table 6). The odds ratio (OR) value at IL-10-819C/T was 4.022. It further indicated that IL-10-819C/T locus (TC plus CC genotype) is probably a very high risk in the occurrence of Deficiency syndrome in HBC patients.

## 4. Discussion 

TCM syndrome classification, also defined as ZHENG differentiation, is the basic concept in the TCM theory. TCM syndrome, a profile of symptoms and signs as a series of clinical phenotypes, plays an important role in understanding the human homeostasis and guiding the applications of TCM treatment. All diagnostic and therapeutic methods in TCM are based on the differentiation of the TCM pattern, and this concept has been used for thousands of years in China [18]. The “Heat,” “Cold,” “Excess,” and “Deficiency” are the four basic syndromes in TCM [19]. In TCM practice, an experiential diagnosis approach has been frequently used to classify Excess, Deficiency, and Deficiency-Excess syndrome in HBC patients. In order to replace the traditional experiential diagnosis, the scientific evidence for TCM syndrome classification is essential, and it would be beneficial to understand the classification and essence of the TCM syndrome. 

IL-10 is an important immunoregulatory cytokine mainly produced by activated T cells, monocytes, B cells, and thymocytes. As an immune response modulator, IL-10 can both stimulate and suppress the immune response [20]. Several polymorphic sites of IL-10 gene promoter region have been described, including three biallelic polymorphisms at positions −1082A/G, −819C/T, and −592A/C from the transcription start site. The IL-10-819C/T C and T alleles were completely in linkage disequilibrium with the IL-10-592A/C A and C alleles, respectively. The −592A allele was exclusively associated with the −1082A allele. These result in three different haplotypes: GCC, ACC, and ATA [21]. It has been reported that IL-10 gene SNP was associated with several diseases such as breast cancer [22], cervical cancer [23], multiple myeloma [24], and gastric carcinoma [25]. Moreover, IL-10 promoter polymorphism was associated with the progression of HBV infection [26]. 

Previous studies have shown that TCM syndrome is associated with gene SNPs. For example, the people with 5-HTTLPR SS genotype polymorphism may be the susceptible population of Excess of liver Yang syndrome [27]. The K allele of ABCA1 gene may be protective factors of phlegm syndrome and blood stasis syndrome in coronary heart disease [28]. The kidney-Yang Deficiency syndrome (KDS) is closely related with special SNP linkage disequilibrium in the intragenic level, and genes within the flanks of these SNPs suggest some essential symptoms of KDS [29]. There was correlation between liver-qi stagnation syndrome and gene polymorphism of tryptophan hydroxylase (TPH) and G-protein*β*3 submit (GNB3) in HBC patients [30]. We have been investigated some cytokine such as TNF-*α*, TGF-*β*_1_, and IL-10 [31] and further found that IL-10 genotype may correlate with TCM syndrome in HBC patients [32]. 

In this study, therefore, to further investigate whether IL-10 genotypes correlated really to TCM syndromes, more samples from different area (Shanghai, Henan and Guangxi in China) were applied, compared to Child-Pugh classification and compensation or decompensation phase. The results showed that IL-10-819C/T locus was significantly correlated to Deficiency syndrome (*P* = 0.031), and IL-10 gene loci (−592A/C, −819C/T, and −1082A/G) were not correlated to either Child-Pugh classification or compensation and decompensation phase in HBC patients. The binary logistic regression analysis showed that the Deficiency syndrome was associated with dry eyes, aversion to cold, IL-10-819C/T and IL-10-1082A/G locus, and OR value at IL-10-819C/T was 4.022. The research results suggested that IL-10-819C/T locus (TC plus CC genotype) might correlate with the risk in the occurrence of Deficiency syndrome in HBC patients. The study provided a proof for TCM syndrome classification, which would be helpful to the TCM clinical diagnosis in HBC patients. 

Though our results showed that IL-10 genotype might correlate with Deficiency syndrome in HBC patients, it is difficult to understand the relationship between IL-10 SNPs and TCM syndromes, while TCM syndrome changes following patient's condition and disease situation. In recent years, following the implementation of Human Genome Project and high throughput Genomic strategies, a large number of human complex diseases associated genetic variants have been identified through Genome-wide association studies (GWAS) [33]. To discover genetic base of TCM syndrome changes as well as other phenotypes of diseases, the GWAS method might provide important clues in future research. 

## 5. Conclusion 

In this study, we identified that IL-10-819C/T locus was significantly correlated to Deficiency syndrome, and the OR value was 4.022, and indicated that HBC patients with the CC genotype plus TC genotype at IL-10-819C/T might correlate with the risk in the occurrence of Deficiency syndrome. 

## Figures and Tables

**Table 1:** Clinical data of 343 HBC patients.

Gender	No (%)
Male	242 (70.55)
Female (%)	101(29.45)
Mean age (yr)	49.57 ± 10.02
Child-Pugh-Turcotte score (%)	
A	240 (69.97)
B	75 (21.87)
C	28 (8.16)
Phase (%)	
Compensation phase	203 (59.18)
Decompensation phase	140 (40.82)
Area (%)	
Shanghai	226 (65.89)
Guangxi	69 (20.12)
Henan	48 (13.99)

**Table 2:** The gene position, polymorphism, primer sequences, and gene frequencies of IL-10 SNPs.

Gene position	Rs number	Polymorphism	Primer sequence	Gene frequencies (%)
IL-10-592 A/C	rs1800872	A/C	F: AAGAGGTGGAAACATGTGCC	22.20
			R: TACCCAAGACTTCTCCTTGC	
IL-10-819 C/T	rs1800871	C/T	F: ATGGTGTACAGTAGGGTGAG	57.70
			R: TTTCCACCTCTTCAGCTGTC	
IL-10-1082 A/G	rs1800896	A/G	F: AGAAGTCCTGATGTCACTGC	15.10
			R: AAGTCAGGATTCCATGGAGG	

**Table 3:** The LDR probes for IL-10 detection.

Gene position	Probes sequence
IL-10-592 A/C	A60-S7-TA: TTTTTTTTAACACATCCTGTGACCCCGCGTGTA
	A60-S7-TC: TTTTTTTTTTTAACACATCCTGTGACCCCGCGTGTC
	A60-S7-TR: -P-CTGTAGGAAGCCAGTCTCTGGAAAGTTTTTT-FAM-
IL-10-819 C/T	A60-S6-TC: TGTACCCTTGTACAGGTGAAGTAAC
	A60-S6-TT: TTTTGTACCCTTGTACAGGTGAAGTAAT
	A60-S6-TR: -P-ATCTCTGTGCCTCAGTTTGCTCACT-FAM-
IL-10-1082 A/G	A60-S5-TA: AACACTACTAAGGCTTCTTTCGGAA
	A60-S5-TG: TTTAACACTACTAAGGCTTCTTTCGGAG
	A60-S5-TR: -P-GGGGAAGTAGGGATAGGTAAGAGGA-FAM-

**Table 4:** Correlation between IL-10 genotypes and Child-Pugh classification or compensation and decompensation phase in HBC patients.

Gene/genotype	Child-Pugh classification			*P*	Phase		*P*
	Class A (%) (*n* = 240)	Class B (%) (*n* = 75)	Class C (%) (*n* = 28)		Compensation (*n* = 203)	Decompensation (*n* = 140)	
IL-10-592 A/C							
AA	104 (43.7)	35 (46.7)	13 (46.4)	0.839	83 (40.9)	69 (50.0)	0.072
AC	108 (45.4)	35 (46.7)	13 (46.4)		95 (46.8)	61 (44.2)	
CC	26 (10.9)	5 (6.7)	2 (7.1)		25 (12.3)	8 (5.8)	
IL-10-819C/T							
TT	114 (47.5)	36 (48.0)	12 (42.9)	0.770	90 (44.3)	72 (51.4)	0.076
CT	103 (42.9)	32 (42.7)	15 (53.6)		89 (43.8)	61 (43.6)	
CC	23 (9.6)	7 (9.3)	1 (3.6)		24 (11.8)	7 (5.0)	
IL-10-1082A/G							
AA	212 (88.3)	65 (86.7)	22 (78.6)	0.340*	182 (89.7)	177 (88.5)	0.710*
AG	27 (11.3)	10 (13.3)	6 (21.4)		20 (9.9)	23 (11.5)	
GG	1 (0.4)	0 (0)	0 (0)		1 (0.5)	0 (0)	

*Between AA and AG + GG of IL-10-1082A/G.

**Table 5:** Correlation between IL-10 genotypes and TCM syndromes in HBC patients.

TCM syndrome type	IL-10-592		*P*	IL-10-819		*P*	IL-10-1082		*P*
	AA	AC + CC		TT	TC + CC		GG	AG + AA	
Excess syndrome	197	29	0.999	111	112	0.600	22	203	0.969
Deficiency-Excess syndrome	41	7	0.735	27	24	0.470	4	49	0.621
Deficiency syndrome	61	8	0.778	23	46	**0.031**	7	56	0.726

Total	299	44		163	180		33	308	

*X ^2^ test.

**Table 6:** Correlation between Excess or Deficiency syndrome and clinical data and IL-10 gene SNPs in HBC patients.

Factors	B	SE	Wald	*P*	OR	95%CI	
						Lower	Upper
Excess syndrome							
Abdominal distension	0.277	0.148	3.509	0.061	1.319	0.987	1.763
Dizzy	0.658	0.203	10.458	0.001	1.931	1.296	2.876
Spider nevus	0.385	0.180	4.594	0.032	1.469	1.033	2.089
Constant	0.173	0.199	0.755	0.385	1.189		
Deficiency syndrome							
Dry eyes	0.448	0.191	5.518	0.019	1.566	1.077	2.276
Aversion to cold	0.605	0.203	8.868	0.003	1.830	1.230	2.725
IL-10-819C/T	1.392	0.442	9.921	0.002	4.022	1.692	9.563
IL-10-1082A/G	−0.903	0.430	4.415	0.036	0.406	0.175	0.941
Constant	−1.163	0.777	2.240	0.134	0.313		

## References

[1] Zhang X, Zhang H, Ye L (2006). Effects of hepatitis B virus X protein on the development of liver cancer. *Journal of Laboratory and Clinical Medicine*.

[2] McMahon BJ (2005). Epidemiology and natural history of hepatitis B. *Seminars in Liver Disease*.

[3] Margolis HS, Alter MJ, Hadler SC (1991). Hepatitis B: evolving epidemiology and implications for control. *Seminars in Liver Disease*.

[4] Lee WM (1997). Hepatitis B virus infection. *The New England Journal of Medicine*.

[5] Weissberg JI, Andres LL, Smith CI, Weick S, Nichols JE, Garcia G (1984). Survival in chronic hepatitis B. An analysis of 379 patients. *Annals of Internal Medicine*.

[6] Liaw YF, Tai DI, Chu CM, Chen TJ (1988). The development of cirrhosis in patients with chronic type B hepatitis: a prospective study. *Hepatology*.

[7] de Jongh FE, Janssen HLA, de Man RA, Hop WCJ, Schalm SW, van Blankenstein M (1992). Survival and prognostic indicators in hepatitis B surface antigen-positive cirrhosis of the liver. *Gastroenterology*.

[8] http://en.wikipedia.org/wiki/Singlenucleotidepolymorphism.

[9] Sachidanandam R, Weissman D, Schmidt SC (2001). A map of human genome sequence variation containing 1.42 million single nucleotide polymorphisms. *Nature*.

[10] Helminen M, Lahdenpohja N, Hurme M (1999). Polymorphism of the interleukin-10 gene is associated with susceptibility to Epstein-Barr virus infection. *Journal of Infectious Diseases*.

[11] Munaka M, Kohshi K, Kawamoto T (2003). Genetic polymorphisms of tobacco- and alcohol-related metabolizing enzymes and the risk of hepatocellular carcinoma. *Journal of Cancer Research and Clinical Oncology*.

[12] Gao QJ, Liu DW, Zhang SY (2009). Polymorphisms of some cytokines and chronic hepatitis B and C virus infection. *World Journal of Gastroenterology*.

[13] Li S, Zhang ZQ, Wu LJ, Zhang XG, Li YD, Wang YY (2007). Understanding ZHENG in traditional Chinese medicine in the context of neuro-endocrine-immune network. *IET Systems Biology*.

[14] Cholongitas E, Papatheodoridis GV, Vangeli M, Terreni N, Patch D, Burroughs AK (2005). Systematic review: the model for end-stage liver disease—should it replace Child-Pugh’s classification for assessing prognosis in cirrhosis?. *Alimentary Pharmacology and Therapeutics*.

[15] Christensen E (2004). Prognostic models including the Child-Pugh, MELD and Mayo risk scores—where are we and where should we go?. *Journal of Hepatology*.

[16] Zhang YX (1994). Dlagnosis, syndrome differentiation of TCM and evaluate the curative effect of liver cirrhosis (tentative scheme). *Chinese Journal of Integrative Medicine*.

[17] Xiao Z, Xiao J, Jiang Y (2006). A novel method based on ligase detection reaction for low abundant YIDD mutants detection in hepatitis B virus. *Hepatology Research*.

[18] Keji C, Hao X (2003). The integration of traditional Chinese medicine and Western medicine. *European Review*.

[19] Jiang WY (2005). Therapeutic wisdom in traditional Chinese medicine: a perspective from modern science. *Trends in Pharmacological Sciences*.

[20] Mocellin S, Marincola FM, Young HA (2005). Interleukin-10 and the immune response against cancer: a counterpoint. *Journal of Leukocyte Biology*.

[21] Eskdale J, Keijsers V, Huizinga T, Gallagher G (1999). Microsatellite alleles and single nucleotide polymorphisms (SNP) combine to form four major haplotype families at the human interleukin-10 (IL-10) locus. *Genes and Immunity*.

[22] Kong F, Liu J, Liu Y, Song B, Wang H, Liu W (2010). Association of interleukin-10 gene polymorphisms with breast cancer in a Chinese population. *Journal of Experimental and Clinical Cancer Research*.

[23] Stanczuk GA, Sibanda EN, Perrey C (2001). Cancer of the uterine cervix may be significantly associated with a gene polymorphism coding for increased IL-10 production. *International Journal of Cancer*.

[24] Zheng C, Huang D, Liu L (2001). Interleukin-10 gene promoter polymorphisms in multiple myeloma. *International Journal of Cancer*.

[25] Wu MS, Huang SP, Chang YT (2002). Tumor necrosis factor-*α* and interleukin-10 promoter polymorphisms in Epstein-Barr virus-associated gastric carcinoma. *Journal of Infectious Diseases*.

[26] Miyazoe S, Hamasaki K, Nakata K (2002). Influence of interleukin-10 gene promoter polymorphisms on disease progression in patients chronically infected with hepatitis B virus. *American Journal of Gastroenterology*.

[27] You JS, Hu SY, Zhang HG (2004). Study on emotion mea surement of liver syndromes in TCM and its correlative study on polymorphism of serotonin (5 HT) transporter gene. *Zhongguo Yi Yao Xue Bao*.

[28] Wu YF, Zhou YC, Zhang XS (2006). Association between traditional Chinese medicine syndrome of coronary atherosclerotic heart disease and polymorphism of R219K of ABCAl gene in Chinese Han male patients. *Zhongguo Zu Zhi Gong Cheng Yan Jiu Yu Lin Chuang Kang Fu*.

[29] Ding WJ, Zeng YZ, Li WH (2009). Identification of linkage disequilibrium SNPs from a Kidney-yang deficiency syndrome pedigree. *American Journal of Chinese Medicine*.

[30] Liu YB, Zhang W, Su SB (2010). Association between liver cirrhosis of hepatitis B cases with Ganqi depression pattern and 5HTTLPRVNTRs,TPH A218C,GNB3 C825T polymorphism. *Chinese Journal of Integrated Traditional and Western Medicine on Liver Diseases*.

[31] Jiang ZL, Zhang H, Su SB, Zhang W, Liu YB (2009). Relationship between gene polymorphisms of TNF-*α*, TGF-*β*1 and IL-10 and posthepatitis B-hepatitic cirrhosis. *World Chinese Journal of Digestology*.

[32] Jiang ZL, Zhang W, Zhang H, Liu YB, Li QY, Su SB (2009). Relationship between gene polymorphisms of interleukin-10 and syndrome types of traditional Chinese medicine in post hepatitis B cirrhosis. *Zhong Xi Yi Jie He Xue Bao*.

[33] Hardy J, Singleton A (2009). Genome-wide association studies and human disease. *The New England Journal of Medicine*.

